# Framework for Accurate Classification of Self-Reported Stress From Multisession Functional MRI Data of Veterans With Posttraumatic Stress

**DOI:** 10.1177/24705470231203655

**Published:** 2023-09-28

**Authors:** Rahul Goel, Teresa Tse, Lia J. Smith, Andrew Floren, Bruce Naylor, M. Wright Williams, Ramiro Salas, Albert S. Rizzo, David Ress

**Affiliations:** 1 Department of Neuroscience, 3989 Baylor College of Medicine, Houston, TX, USA; 2 Department of Psychology, 14743 University of Houston, Houston, TX, USA; 3 20116 Center for Translational Research on Inflammatory Diseases, Michael E. DeBakey VA Medical Center, Houston, TX, USA; 4 Department of Electrical and Computer Engineering, 12330 University of Texas at Austin, Austin, TX, USA; 5 Department of Psychiatry and Behavioral Sciences, 3989 Baylor College of Medicine, Houston, TX, USA; 6 20986 The Menninger Clinic, Houston, TX, USA; 7 Institute for Creative Technologies, 5116 University of Southern California, Los Angeles, CA, USA

**Keywords:** functional magnetic resonance imaging, algorithm design and analysis, machine learning algorithms, behavioral sciences, psychiatry, mental disorders, posttraumatic stress disorder (PTSD), veterans

## Abstract

**Background:** Posttraumatic stress disorder (PTSD) is a significant burden among combat Veterans returning from the wars in Iraq and Afghanistan. While empirically supported treatments have demonstrated reductions in PTSD symptomatology, there remains a need to improve treatment effectiveness. Functional magnetic resonance imaging (fMRI) neurofeedback has emerged as a possible treatment to ameliorate PTSD symptom severity. Virtual reality (VR) approaches have also shown promise in increasing treatment compliance and outcomes. To facilitate fMRI neurofeedback-associated therapies, it would be advantageous to accurately classify internal brain stress levels while Veterans are exposed to trauma-associated VR imagery. **Methods:** Across 2 sessions, we used fMRI to collect neural responses to trauma-associated VR-like stimuli among male combat Veterans with PTSD symptoms (N = 8). Veterans reported their self-perceived stress level on a scale from 1 to 8 every 15 s throughout the fMRI sessions. In our proposed framework, we precisely sample the fMRI data on cortical gray matter, blurring the data along the gray-matter manifold to reduce noise and dimensionality while preserving maximum neural information. Then, we independently applied 3 machine learning (ML) algorithms to this fMRI data collected across 2 sessions, separately for each Veteran, to build individualized ML models that predicted their internal brain states (self-reported stress responses). **Results:** We accurately classified the 8-class self-reported stress responses with a mean (± standard error) root mean square error of 0.6 (± 0.1) across all Veterans using the best ML approach. **Conclusions:** The findings demonstrate the predictive ability of ML algorithms applied to whole-brain cortical fMRI data collected during individual Veteran sessions. The framework we have developed to preprocess whole-brain cortical fMRI data and train ML models across sessions would provide a valuable tool to enable individualized real-time fMRI neurofeedback during VR-like exposure therapy for PTSD.

## Introduction

Veterans return from the wars in Iraq and Afghanistan with high rates of posttraumatic stress disorder (PTSD), with concomitant long-term costs to themselves, their families, and society at large.^1,2^ The Department of Veteran's Affairs (VA) has responded to these concerns by offering evidence-based psychotherapy treatments, including exposure therapies, specifically designed for Veterans with PTSD. However, despite their well-established efficacy,^3,4^ many Veterans drop out of exposure therapies or continue to meet clinical criteria for PTSD after completing them.^5–8^ Hence, there is a need for additional novel treatment interventions.

Functional magnetic resonance imaging (fMRI) neurofeedback (eg, Refs^9–11^) and virtual reality (VR) (eg, Refs^12,13^) have independently shown promise in producing improvements for exposure-based therapies. For example, fMRI neurofeedback, a procedure relying upon the blood oxygen level–dependent imaging signal in the left amygdala, was able to reduce anxiety.^14^ However, the broader use of the cortex may be a better approach for PTSD fMRI neurofeedback, since PTSD treatment may improve functioning in several neural networks and brain regions that span the cortex, including the salience network associated with threat detection, executive function network, emotion regulation areas, and brain circuits involved in contextual processing used for threat discrimination.^15^ A second treatment approach, VR, may increase treatment effectiveness by improving a Veteran's ability to confront traumatic events with richer sensory detail than in classic “imaginal” exposure, that is, mental imagery.^16^ In VR, the clinician may decrease avoidance by controlling the stimuli in the scene.^17^ For combat-related PTSD, software applications that present visual and auditory cues relevant to combat zones may be a helpful tool.^18^

Overall, both fMRI neurofeedback and VR approaches have shown promise but have primarily been used independently from each other. Emerging research outside the area of PTSD^19,20^ suggests that combining them could have a synergistic effect. An attractive long-range goal, therefore, would be the use of machine learning (ML) to classify real-time whole-brain cortical fMRI data, which match self-reported stress levels, while the Veteran is exposed to different levels of stress-inducing stimuli using VR. If successful, such information could be helpful to a clinician, or as feedback to the Veteran to help them react appropriately to traumatic stimuli. To perform such studies, the first step is to demonstrate that we can decode cortical variables inherent to the subject rather than the stimuli across different fMRI timepoints. The current feasibility study presents a framework for training different ML models across 2 sessions using the entire activation data of the cerebral cortex for each subject. Preprocessing was limited to a subset of operations consistent with our eventual goal of real-time analysis. To improve data fidelity, we used high-resolution fMRI that focused explicitly on gray matter. We then blurred these data along with the cortical manifold to reduce the dimensionality of the data while maximizing information relevant for decoding. The resulting framework provides accurate stress classification, and the decoding computations can be executed with sufficient speed to enable real-time application in the future.

Although brain state classification from fMRI data is challenging,^21–23^ our group has demonstrated accuracy in predicting a complex internal brain state, the number of viewed characters, with 2 ML algorithms: support vector machine (SVM) and artificial neural network (ANN).^24^ Support vector machine has been used to distinguish high and low cognitive load,^25^ or visual object recognition.^26^ A variant of SVM, support vector regression (SVR), is an alternative approach used in some studies, for example, to predict the subjective experience of Veterans playing a VR game involving search tasks,^27^ or a visuospatial task,^28^ and in our preliminary work in which we predicted the difficulty level of a game played in a VR environment. The multilayer perceptron (MLP), which is an extension of the ANN, has been used to distinguish individuals with schizophrenia from controls^29^ and to identify decision-making voxels in a visual task.^30^

The current study exposed Veterans with combat-related PTSD symptoms to variably stressful VR-like stimuli. Cortical responses were collected using whole-brain fMRI and analyzed with ML techniques. We tested whether ML, applied to the downsampled gray-matter fMRI data, could reliably classify self-reported stress intensity levels in Veterans.

## Methods

### Study Design

Eight male combat Veterans from Operation Enduring Freedom (OEF)/Operation Iraqi Freedom (OIF)/Operation New Dawn (OND) diagnosed with PTSD were recruited from Michael E. DeBakey VA Medical Center in Houston, TX. Veterans were initially included in the study if they saw combat in the wars in Iraq and Afghanistan and met the criteria for PTSD using the Clinician-Administered PTSD Scale for DSM-5, CAPS-5.^31^ Veterans who did not meet the criteria for PTSD, could not provide consent, or were unable to be scanned (eg, implantable/external electronic devices, and shrapnel) were excluded. Informed consent was obtained under a protocol approved by the Baylor College of Medicine Institutional Review Board.

Veterans participated in an initial 20 min MRI session to record brain anatomy. During this session, a high-resolution T1-weighted structural reference volume was obtained using a high-resolution MP-RAGE sequence: TI = 900 ms, TR = 2600 ms, flip angle 9°, cubic 0.7 mm voxels, whole-brain coverage, on a Siemens 3 T Prisma scanner using a 64-channel head coil.

Next, the study participants completed 2 fMRI sessions. Aside from 1 subject scanned early in the project during pilot work, the 2 sessions were collected between 3 and 13 days apart; the mean (standard deviation [SD]) difference was 7.7 (3.9) days. fMRI data were acquired on the same scanner with a T2*-weighted echo-planar imaging sequence (TE = 30 ms, Ernst flip angle) that utilized both 2:1 GRAPPA acceleration and 3:1 simultaneous multislice acceleration,^32,33^ yielding 2 mm cubic voxels on 63 slices at TR = 1.5 s over a 192 mm field-of-view. An inplane set of T1-weighted structural images were also obtained using a FLASH sequence (minimum TR and TE, 1 mm pixels) on the same slice prescription as the fMRI, at the end of each functional session. Inplane images were used to register the fMRI data with each Veteran's high-resolution anatomy. The study protocol described was identical during both fMRI sessions. Individualized VR-like sequences were created for each fMRI session.

**Figure 1.**
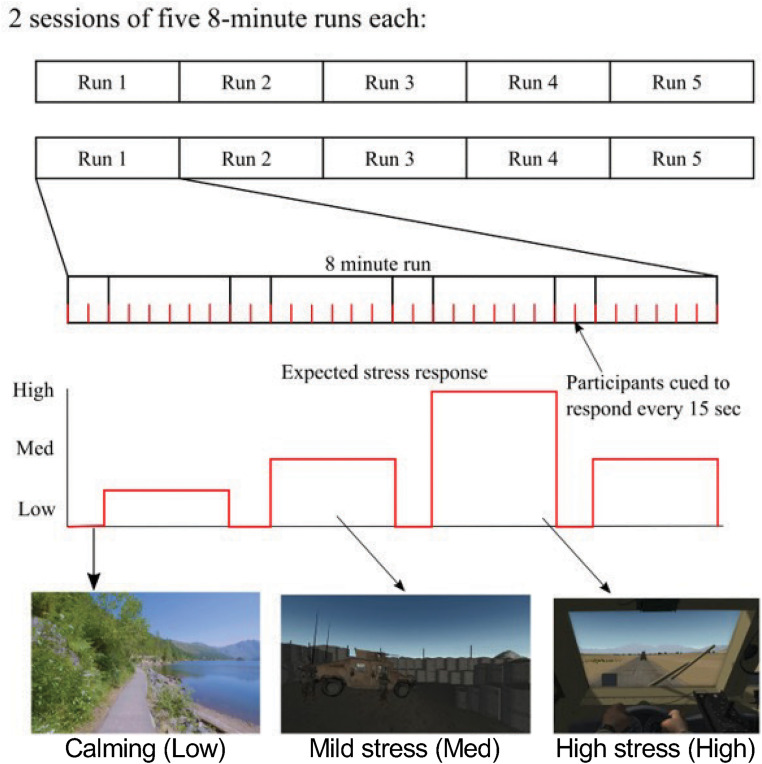
Individualized VR sequences included 2 fMRI sessions of five 8-min runs with a break of 1 to 2 min between each run. Veterans received an audio cue every 15 s to provide their subjective stress level on a 1 to 8 scale (top). VR-like sequences included alternating calming and stress-inducing stimuli such that Veteran stress levels varied across each run (middle). Calming, mildly stressful, and stressful stimuli were created based on clinical interviews with each Veteran (bottom). VR, virtual reality.

Variably stressful VR-like stimuli were created using BraveMind,^18,34^ a VR environment application designed for the OEF/OIF/OND Veteran cohort. The development of individualized VR-like stimuli was informed by an in-vivo hierarchy procedure, based upon past work,^35–38^ developed collaboratively between each Veteran and the clinician before the first fMRI session. Starting with each Veteran's self-identified “worst” combat-related traumatic event, the clinician and Veteran reviewed relevant BraveMind VR stimuli to develop a hierarchy of 8 stress-inducing scenarios ranging from the least (1) to the most (8) stressful. Calming stimuli (ie, level 1) were based on individual Veteran preferences (eg, beach, nature hike; Figure 1). Low-stress stimuli (eg, levels 2-4) typically featured scenes such as a secured military operating base. High-stress stimuli (eg, levels 5-8) included nuanced environments specific to each Veteran's combat-related traumatic events. For example, improvised explosive devices were depicted using BraveMind software. Individualized 8 min audio/video sequences were created for each Veteran to probe a wide range of stress responses. Sequences consisted of multiple 60 or 90 s duration clips of variably stressful stimuli and 30 or 60 s duration clips of calming stimuli. Veterans viewed variably stressful clips, followed by rest periods with a calming stimulus.

In total, ten 8 min personalized sequences were created for each Veteran. Five sequences were played during the first fMRI scanning session, and the remaining 5 were played during the second fMRI scanning session (Figure 1). During fMRI data collection, Veterans viewed stimuli on an MRI-compatible LCD monitor (BOLDscreen 32, Cambridge Research Systems) positioned immediately behind the scanner bore and viewed through a first-surface mirror. Audio stimuli were delivered using a pair of MRI-compatible earphones (Sensimetrics).

### Measurements

A self-administered survey assessing trauma history and demographic information was conducted to collect information from all participants. Lifetime traumatic event exposures were collected with the Life Events Checklist for DSM-5, LEC-5.^39^ PTSD symptomatology was collected via a clinical interview with the CAPS-5.^31^

An 8-point scale was utilized to assess subjective stress responses to the VR-like stimuli. Veterans were given two 4-button MR-compatible response pads (Current Designs), one for each hand. They were trained to provide their stress responses by pressing 1 of the 8 buttons (1 being the least stressful and 8 being the most stressful). An audio tone occurred every 15 s to cue the subject to report the stress level. Since the fMRI repetition time (TR) was 1.5 s, the subjective stress response was obtained every 10 brain-volume acquisitions (frames). Video clips created for inducing different levels of stress were 30, 60, or 90 s long. To elicit a consistent stress level, there was relatively little variation in the intensity within each video clip. Thus, whenever a Veteran reported a particular stress level, it was assumed to be representative of the stress response over the preceding 10 frames (15 s). Pulse and respiration were not reliably measured in the current study due to hardware problems.

### MRI Analysis

Automatic cortical segmentation and surface extraction were performed on the structural reference volume using “expert options”^40^ in the FreeSurfer package^41^ to enable processing at the native resolution (0.7 mm) of the reference volume. The segmentation also yielded surface representations of each brain at the gray–white interface.^42^

The inplane anatomical volumes were skull-stripped and normalized in the same fashion as the first stage of the automatic cortical segmentation and surface extraction of reference volumes. To compensate for head motion, the processed inplane volumes were then affinely registered to the reference volumes using a method based on robust statistics to detect outliers and remove them from the registration.^43^

**Figure 2.**
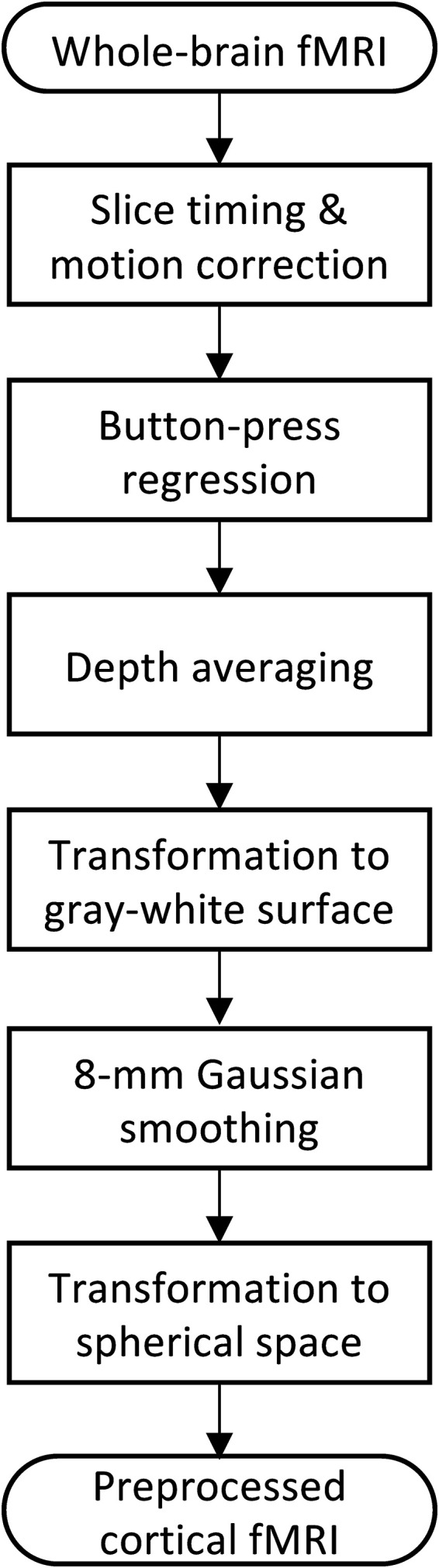
Preprocessing and transformation of the collected whole-brain fMRI data into cortical fMRI data, which were then used in machine learning (ML) analysis.

The steps used to preprocess fMRI data are shown in Figure 2. Only a subset of standard preprocessing operations was used because of our eventual application of these methods to real-time therapy. These steps eventually formed a comparatively low-dimensional feature set (∼5100 features) for subsequent ML analyses while still providing the gray-matter-activation information from the whole cortex. Because our preprocessing steps were more limited than standard practice, we later tested the influence of the missing operations on the data quality. See Supplemental Material (SM1) for the detailed MRI preprocessing steps.

### Machine Learning Analyses

We explored the effectiveness of 3 different supervised ML algorithms in predicting self-reported stress responses using cortical surface voxels as features: SVM classifier,^24,26,44,45^ SVR,^27,28^ and MLP classifier,^29^ which is a kind of deep learning (DL) approach utilizing multiple feed-forward ANN models. We used self-reported stress responses as the ground truth, as is commonly done in therapeutic settings.^36,46,47^ Accurate classification of self-reported responses across the 8-point scale of subjective stress provided a measure of how the Veteran was feeling internally. The primary ML challenge was to decode across 8 levels of saliently different inherent brain states rather than classify any external stimuli or sensory task. See Supplemental Material (SM2) for ML-processing details.

## Results

### Descriptive Statistics

The sample consisted of 8 OEF/OIF/OND Veterans, all identifying as male, aged 43.9 ± 10.5 years (mean ± SD). The mean CAPS-5 score was 38.8 ± 8.4, indicating moderate-to-severe PTSD symptomatology.^48^ Further Veteran characteristics are detailed in Table 1. The range of stress responses obtained from the Veterans during scan sessions is shown in Table 2.

**Table 1.** Demographics of Veterans.

Veteran characteristics	N = 8
Race/Ethnicity	
Black/African American	50.0% (4)
White	37.5% (3)
Hispanic	12.5% (1)
Age	43.9 (10.5)
Gender (Male)	100% (8)
Marital status	
Married	37.5% (3)
Never married	25.0% (2)
Divorced	25.0% (2)
Separated	12.5% (1)
Branch of military service	
Army	87.5% (7)
Marines	12.5% (1)
Navy	12.5% (1)
Years in military service	12.9 (8.7)
Combat locations	
Iraq	87.5% (7)
Afghanistan	25.0% (2)
Other (eg, Kuwait)	37.5% (3)
Trauma event exposures	
Average traumatic event types	12.9 (2.2)
Fire or explosion	100.0% (8)
Transportation accident	87.5% (7)
Serious accident	62.5% (5)
Toxic substance exposure	75.0% (6)
Physical assault	100.0% (8)
Assault with a weapon	87.5% (7)
Sexual assault	25.0% (2)
Unwanted sexual experience	50.0% (4)
Combat	100.0% (8)
Captivity	12.5% (1)
Life-threatening illness or injury	87.5% (7)
Human suffering	87.5% (7)
Sudden violent death	100.0% (8)
Sudden accidental death	87.5% (7)
Caused serious injury to someone	75.0% (6)
Other traumatic or stressful event	50.0% (4)
PTSD symptoms	
Average PTSD symptom severity	38.8 (8.4)
Reexperiencing	9.4 (2.8)
Avoidance	4.6 (2.1)
Negative alterations in cognitions and mood	12.5 (5.0)
Arousal and reactivity	12.3 (2.4)

Mean (SD) or % (n). Trauma event exposures from Life Events Checklist for DSM-5.^39^ Mean presession PTSD symptom scores from the Clinician-Administered PTSD Scale for DSM-5.^31^

**Table 2.** Range of Self-Reported Stress Responses for Each Veteran Across the 2 fMRI Sessions.

Veteran	1	2	3	4	5	6	7	8
Session 1	1-7	1-8	1-6	1-8	1-8	1-7	1-4	1-8
Session 2	1-5	1-8	1-8	1-7	1-8	1,3-8	1-5	1-6

Each Veteran completed 2 fMRI sessions, an average of 7.7 days apart. The Veterans were provided with an 8-point scale to report their subjective stress levels throughout each session. Table displays a range of subjective stress responses provided by each Veteran during each fMRI session.

### Results of ML Analyses

**Figure 3.**
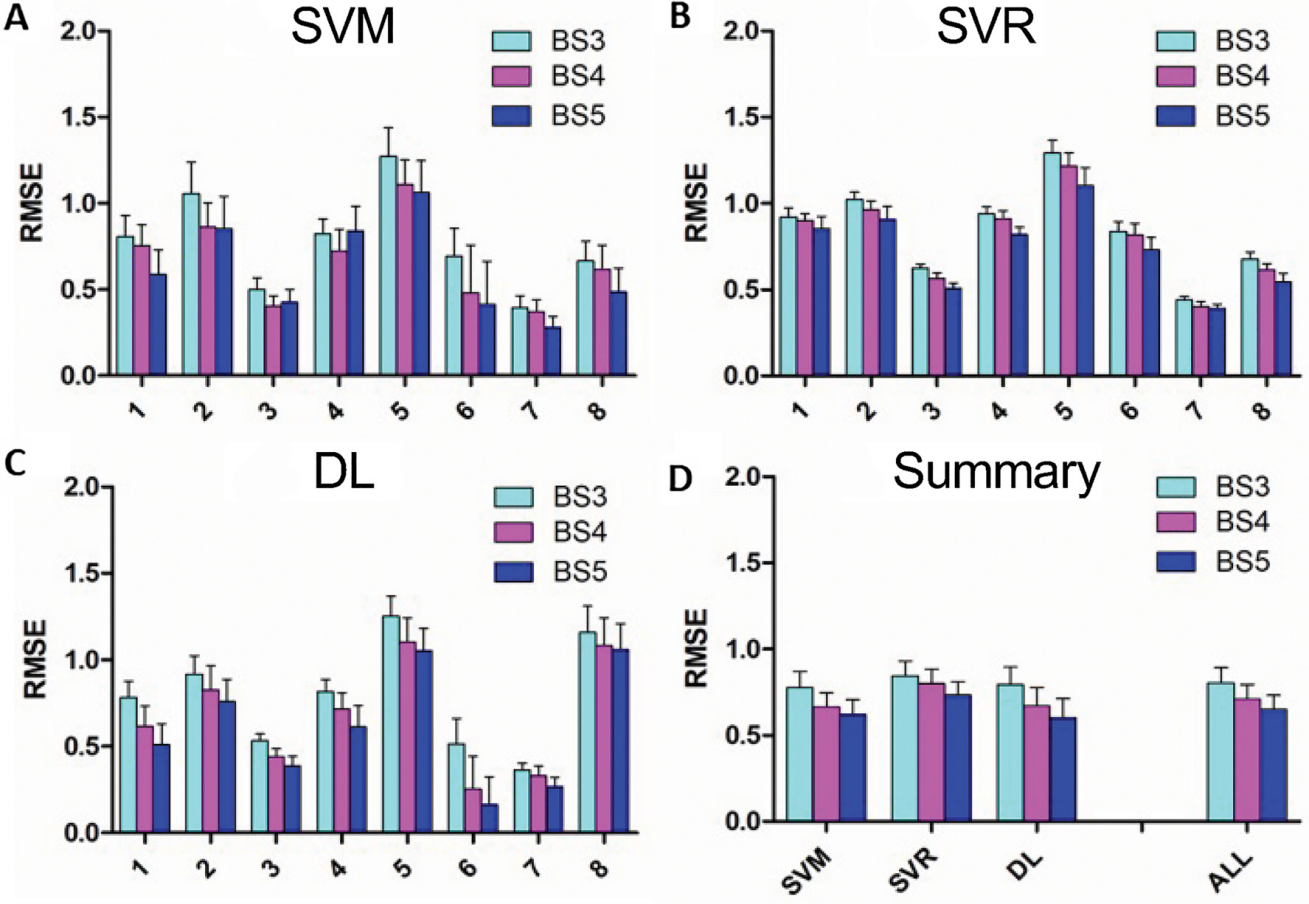
(A-C) Mean root mean square error (RMSE) values for 3 different ML models for each of the 3 different block size combinations, for each of the 8 Veterans. (D) Summary of RMSE across all Veterans for each of the 3 different ML models and for the 3 models combined for each of the 3 block sizes. Error bars represent standard deviation for A to C and standard error across Veterans for D. SVM, support vector machine; SVR, support vector regression; DL, deep learning; BS#, block size of # frames; ML, machine learning.

The mean and SD of the RMSE across the 25 iterations for each Veteran for the 3 ML models and the 3 block sizes are shown in Figure 3A to C. For most Veterans, results show RMSE <1 for all 3 ML methods, with the smallest error typically obtained using the DL approach. Support vector machine performed better than SVR across all Veterans and all the 3 block-averaging schemes (*P* < .001). In contrast, DL performed better than both support vector methods (*P* < .001) for all Veterans except Veteran 8.

As shown in Figure 3D, the mean and standard error (SE) of RMSE values for the different ML models (SVM, SVR, and DL) were averaged across all 8 Veterans, for each of the 3 block sizes. The ALL group on the right side of Figure 3D demonstrates results after averaging the results of SVM, SVR, and DL for each Veteran first and then combining that average across all Veterans for the 3 block sizes. For these data, the RMSE of BS5 was better than that of BS4 (*P* = .002), and BS4 was better than BS3 (*P* < .001), across all 8 Veterans.

In terms of optimal features, for each Veteran, we first took the median of optimal features across the 3 block sizes for each of the 3 ML models. The results showed that for SVM, 4700 features were optimal for 7 of the Veterans, and 2400 were optimal for 1 Veteran; for SVR, 4700 features were optimal for 7, and 1600 features were optimal for 1 Veteran; and for DL, 4700 features were optimal for 6, and 2400 features were optimal for 2 Veterans. Additionally, since DL usually performed better than the two support vector techniques for most Veterans, we also looked at median optimal features for the DL approach across all Veterans and block sizes. For that, 4700 features were optimal.

Regarding accuracy, SVM was significantly (*P* < .001) better than DL across all 8 Veterans and all 3 block-averaging schemes. Specifically, the mean ± SE accuracy for SVM for BS3, BS4, and BS5 was 85.5 ± 2.1%, 88.0 ± 2.0%, and 88.9 ± 1.9%, respectively, whereas that of DL was 80.0 ± 4.2%, 82.0 ± 4.3%, 84.6 ± 4.1%, respectively.

### Results of Permutation Testing

**Figure 4.**
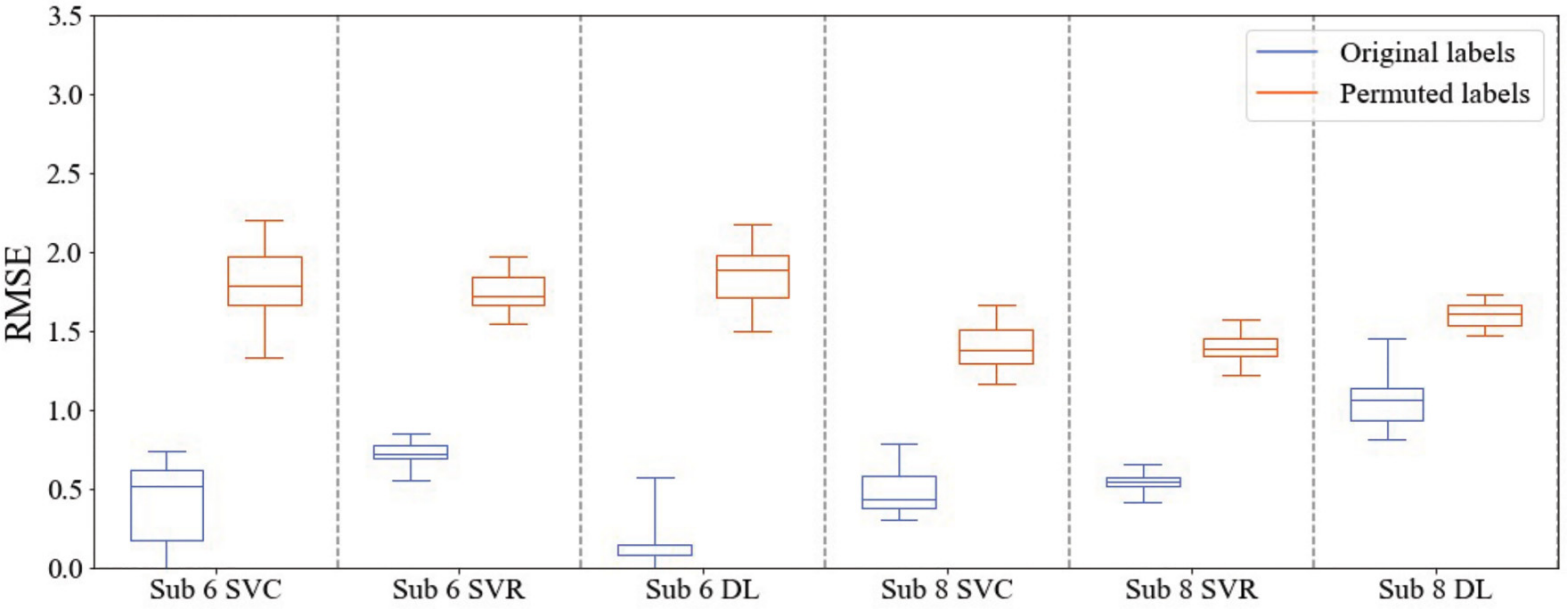
Box plots illustrating the results of the permutation tests for 2 of the Veterans. The blue boxplots represent the RMSE values for the ML models run with the original labels, while the orange boxplots represent the RMSE values for the ML models run with permuted labels. ML, machine learning.

Further, to test the statistical significance of the performance of the 3 ML models, we ran permutation tests.^49^ Using the best block-averaging size (BS5), the ML models were run 25 times with the class labels randomly permuted. The results of the permutation tests for 2 Veterans (#6 and #8) are shown in Figure 4, while the results for the remaining 6 Veterans are in Supplemental Material (Figure SM3). The blue boxplots in Figure 4 show the RMSE values for the respective ML model run with the original, accurate labels, while the orange boxplots show the RMSE values for the ML model run with permuted labels. For all 3 ML models for the Veterans, the original labels’ RMSE values are significantly smaller than those of the permuted labels. In addition, the distribution of the RMSE values for the original labels does not overlap with that of the permuted labels. Thus, we can conclude that all 3 ML models found a significant class structure and that the cortical fMRI data and the class labels are dependent.

### Results of Testing With Additional fMRI Preprocessing Steps

Lastly, we tested the addition of 3 fMRI preprocessing steps (motion parameter regression, temporal high-pass filtering, and Wiener filtering) for 3 representative participants (Veterans 3, 6, and 8), using methods described in Floren et al.^24^ Note that the Wiener filtering was used specifically to remove hemodynamic delay, not as a form of temporal smoothing. We added these 3 preprocessing steps in succession and tested the performance of SVM with a block size of 5. The resulting RMSE values are shown in Table 3. The classification accuracy was usually worse with additional preprocessing.

**Table 3.** Classification Accuracy With the Addition of Standard fMRI Preprocessing Steps.

Veteran	Additional preprocessing	RMSE mean (SD) for different iterations
Veteran 3	None (base case)	0.490 (0.094)
	Motion regression	0.525 (0.099)
	High-pass filtering, motion regression	0.604 (0.082)
	High-pass filtering, motion regression, Wiener filtering	1.029 (0.102)
Veteran 6	None	0.492 (0.235)
	Motion regression	0.783 (0.231)
	High-pass filtering, motion regression	1.043 (0.168)
	High-pass filtering, motion regression, Wiener filtering	0.686 (0.197)
Veteran 8	None	0.786 (0.159)
	Motion regression	0.635 (0.177)
	High-pass filtering, motion regression	1.162 (0.141)
	High-pass filtering, motion regression, Wiener filtering	0.966 (0.158)

## Discussion

This feasibility study describes and tests a framework in which fMRI data are sampled specifically from the gray matter with 2-mm isotropic voxels, then blurred along with the cortical surface in a fashion that reduces noise and dimensionality while maintaining gray-matter specificity. After that, we examined an ML approach to classify 8 salient levels of an inherent brain state (self-reported stress responses) from cortical fMRI data. Veterans with combat-related PTSD were exposed to different levels of trauma-associated VR-like stimuli. We applied 3 ML algorithms to the downsampled cortical fMRI data, separately for each Veteran, to build Veteran-specific ML models to predict stress responses.

The results are very promising for future clinical applications. Generally, whole-brain cortical fMRI data are too high dimensional to permit efficient ML training, so some form of feature reduction is necessary. Here, we utilized an anisotropic blurring scheme that delineates gray matter but blurs and downsamples the data appropriately across the gray-matter ribbon. Because brain function is commonly clustered into centimeter-scale regions, for example, the functional atlas elements established by the Human Connectome Project,^50^ this approach provides an effective means of feature reduction. This whole-cortex downsampled data enabled ML to predict inherent brain states at eight salient levels instead of binary classification of stimuli or tasks. The best-performing ML algorithm classified the Veterans’ stress responses with an RMSE of 0.6 ± 0.1 (mean ± SE) across all 8 Veterans. For most Veterans, results demonstrated RMSE < 1.0. Even for the Veteran with the worst performance in our models (Veteran 5), we could obtain a mean RMSE of 1.05 with DL-BS5, which could still be helpful for a real-time therapeutic application. Typically, we observed improvements in performance as block size increased from BS3 to BS5 for all 3 ML approaches. We also demonstrated that while DL training is computationally intensive, decoding was fast (see SM2). Even using the overall slowest approach, SVR, it would only take approximately 2.5 ms to decode a new sample. Overall, our new proposed framework of preprocessing and ML training and decoding shows promise as a tool for real-time fMRI neurofeedback, which could be applied during VR exposure therapy for PTSD.

We tested the feasibility of decoding self-reported stress from neural signals measured from cortical fMRI as a potential tool for future use in real-time neurofeedback, in contrast to the previous region-of-interest-based approaches, for example, Refs^14,51^ We optimized for 3 different numbers of features (1600, 2400, and 4700). We found that using 4700 features proved optimal across all Veterans, ML models, and block-averaging schemes. We reasoned that more complex classification functions could be learned because cortical fMRI offers a much larger feature set. We found that DL outperformed both support vector methods (in terms of RMSE), which performed similarly for all but one Veteran.

Future research should replicate and extend this work. For example, future work may utilize the output of several ML models and block-averaging schemes for real-time applications. For later real-time application, we intend to test averaging temporally adjacent 3, 4, or 5 samples, using a sliding window approach, to reduce the effects of fMRI noise and physiological artifacts.^14,52^ This work may also be extended via comparisons to nonclinical controls. It may also be beneficial to examine whether performance can be improved with more complex DL algorithms like long short-term memory/convolutional neural network, which learns from time sequence and spatial distribution of features, respectively, as well as transfer learning approaches that are gaining popularity.^23,53^ Therapeutic approaches utilizing exposure, for example, Refs,^3,46,54–57^ and neurofeedback, for example, Refs,^58,59^ techniques for combat-related PTSD may also benefit from research that helps identify which Veterans may benefit most from real-time fMRI neurofeedback with VR as a therapeutic alternative. Indeed, trauma researchers have been asking “what works for whom” to identify treatments that may exhibit the most benefit in specific patient subgroups.^60,61^ Given that the ML models were unique to each Veteran, the current study points the way toward individualized treatment for PTSD. Additionally, collecting physiological variables (eg, respiration) in future work may increase our understanding of this line of work. It is possible that the classification performance may further improve by developing additional ways to remove possible physiological “noise,” or using sensitivity analyses^24^ to examine which features were most predictive of Veterans’ stress responses. This may replicate and extend the extant PTSD neuroscience literature, for example, Refs,^59,62,63^ and inform and extend the efficacy of current evidence-based psychotherapy treatment for PTSD.^64^

The current feasibility study has several strengths. First, it presents a novel framework for preprocessing and ML training of high-dimensional fMRI data, which may benefit emerging neurofeedback therapeutic approaches for OEF/OIF/OND Veterans. Second, we used well-defined inclusion criteria for OEF/OIF/OND Veterans, reducing heterogeneity. Third, we studied different ML algorithms and parameters. Therapeutically useful RMSEs were obtained for all methods, demonstrating the robustness of the cortical classification approach. Fourth, while group-averaged classification RMSEs were excellent, high-quality results were also observed for most individuals. Finally, ML algorithms were trained on each Veteran's data, generating completely individualized models that necessarily incorporated unique trauma histories and individual characteristics.

We observed that several preprocessing steps, motion regression, high-pass filtering, and Wiener filtering, did not improve classification performance, an interesting result. It may be that useful classification information is contained in motion-induced intensity artifacts. Perhaps subjects engage in stereotypical head motions during periods of stress. Slow changes in image intensity, removed by high-pass filtering, could reflect hemodynamic or metabolic changes associated with stress states, also providing useful information. Finally, Wiener filtering serves to align the stress state and the fMRI data more precisely. In 2 of the 3 subjects, the filtering slightly improved the classification performance. However, the slow, 15 s sampling of the stress state may render such a correction relatively unimportant. Altogether, the lack of improvement associated with these preprocessing steps is good news for eventual real-time applications of our classification methods, but a more detailed evaluation of the preprocessing workflow will be needed to find an optimal approach.

However, the study has some limitations. First, our results indicated some variability across Veterans, making it possible that this approach may not apply to all Veterans with combat-related PTSD. Second, we did not collect data on individual Veteran exposure to video games and/or VR environments before the current study. One of the Veterans reported extensive gaming experience and did not find the VR-like visual stimuli stressful. However, he did note that the VR audio component was stress-inducing. Data regarding gaming history may be utilized as a covariate in future work. Third, Veterans in the current study noted limitations in BraveMind software (eg, lack of fear response in other soldiers, limited vehicle options) that reduced the realism of the VR-like environment. Thus, we were limited in how well the VR-like stimuli were able to match specific combat-related traumatic experiences reported by the Veterans. Improved software in the future may allow for increased immersion in the VR environment, including the ability to enable both the clinician and Veterans to increase control while in the VR environment. Fourth, although care was taken to regress out button press responses that Veterans provided during the training session, as that is something that will not be available during therapy sessions, it is possible that some other nuances of the training sessions, such as the duration and force used to press the button or the presence of the audible cue, could also have a slight influence on the fMRI data and were not wholly removed. Fifth, given the partial success of some other neurofeedback approaches,^14,52^ it is possible that including subcortical information, especially from the amygdala, may also improve ML classification. However, we chose to delimit our consideration to the cortex because it permitted a spatial downsampling approach to generate features used in ML. Finally, we acknowledge that fMRI-based neurofeedback will remain an expensive option in terms of scalability. However, we believe that our results suggest that such stress classification requires interrogation of the whole cortex compared to more straightforward measurements of stress such as pulse, respiration, and galvanic skin response that mostly interrogate the autonomic nervous system.

## Conclusions

In clinical treatment, many Veterans drop out of exposure therapy or choose other treatments because of their difficulty tolerating the stress of engaging with their traumatic past.^6–8^ To match this difficulty, we propose developing individualized therapeutic approaches utilizing real-time fMRI feedback—a highly desirable yet challenging goal. Our method presents VR-like stimuli tailored to the Veteran's level of distress. The current feasibility study is a critical step to enable the combination of both real-time fMRI neurofeedback and VR for Veterans with combat-related PTSD. We propose a framework of preprocessing fMRI data into lower-dimensional space that provides an adequate sampling of the whole of the cerebral cortex and then applying ML algorithms. Our results suggest that accurate prediction of 8 saliently different levels of an inherent brain state (self-reported stress responses) obtained by presenting stimuli in a realistic environment using VR is feasible. This new method may provide a valuable tool for individualized therapeutic interventions utilizing real-time fMRI neurofeedback to optimize the VR presentation for people undergoing exposure therapy for PTSD.

## Supplemental Material

sj-docx-1-css-10.1177_24705470231203655 - Supplemental material for Framework for Accurate Classification of Self-Reported Stress From Multisession Functional MRI Data of Veterans With Posttraumatic Stress Click here for additional data file. Supplemental material, sj-docx-1-css-10.1177_24705470231203655 for Framework for Accurate Classification of Self-Reported Stress From Multisession Functional MRI Data of Veterans With Posttraumatic Stress by Rahul Goel, Teresa Tse, Lia J. Smith, Andrew Floren, Bruce Naylor, M. Wright Williams, Ramiro Salas, Albert S. Rizzo and David Ress in Chronic Stress
