# Supplementary material for: Framework for Accurate Classification of Self-Reported Stress From Multisession Functional MRI Data of Veterans With Posttraumatic Stress
Source: Chronic Stress (Thousand Oaks). 2023 Sep 28;7:24705470231203655. doi: 10.1177/24705470231203655 (PMC10540591; doi:10.1177/24705470231203655)
Supplement: sj-docx-1-css-10.1177_24705470231203655 - Supplemental material for Framework for Accurate Classification of Self-Reported Stress From Multisession Functional MRI Data of Veterans With Posttraumatic Stress [file sj-docx-1-css-10.1177_24705470231203655.docx]

**Supplementary Material**

*SM1. Detailed methods of MRI processing*

Simultaneous slice-timing and motion correction was performed on each of the functional scans[^1^](#_ENREF_1). A rigid-body registration was performed between scans to align each frame to the last volume, i.e., the frame closest in time to the structural inplane[^2^](#_ENREF_2). The functional data were then approximately aligned to the structural reference volume using the previously calculated registration with the inplane anatomical data. A boundary-based registration technique was then used to fine-tune the registration of the functional data to the structural reference volume[^3^](#_ENREF_3). Button presses (due to responses that the Veterans provided) were then regressed using a standard general linear modeling approach by assuming each button press to be an impulse and convolving the train of button presses with a standard hemodynamic response function (HRF). In the next step, the functional data were projected onto the extracted cortical surfaces of the left and right hemispheres by averaging between white and pial surfaces along the surface normal. To minimize partial volume effects, values were only averaged between 20% and 80% of the distance between the white and pial surfaces along the normal on the white surface[^4^](#_ENREF_4). The functional data were smoothed along the surface with a Gaussian filter (8-mm full-width-at-half-maximum) and then projected onto an icosahedron (order 4) with uniform spacing of vertices in the spherical template space[^5^](#_ENREF_5). We experimented with several different smoothing values and icosahedron order numbers (which determines the density of vertices on the sphere, i.e., the resolution of the data), to find parameters that sufficiently reduce the dimensionality of the data while still retaining as much information as possible. More details on that can be found in our previous preliminary work[^6^](#_ENREF_6).

We also tested the addition of three commonly-used fMRI preprocessing steps to our pipeline: 1) a temporal nonlinear high-pass filter (using the *fslmaths* command in the FSL package) at 0.001 Hz to remove low-frequency signal drifts, 2) regression of motion parameters and their first derivatives to correct for noise in the signal due to small head movements, and 3) Wiener filtering with a signal-to-noise ratio (SNR) of 5 to deconvolve the fMRI signal with the standard HRF. The high-pass filter and motion parameter regression steps were performed after slice-timing and motion correction and before button press regression. The Wiener filtering step was performed after transformation to spherical template space.

**Fig. SM1**. Scheme used in the ML analysis to train and test the models.

*SM2. Detailed methods of Machine learning*

All ML models were trained separately for each Veteran, using data from both fMRI sessions. Each session consisted of five 8-minute runs, resulting in 40 minutes of data (i.e., 1600 frames were collected per session per Veteran). First, we combined all the data collected from the two sessions, resulting in a total of 3200 frames, with ~5100 features (smoothed surface samples as explained in the previous section) and stress labels typically varying from 1-8 for most Veterans. It is known that the sluggish hemodynamic response will introduce temporal correlations between consecutive frames[^7^](#_ENREF_7). Consequently, instead of using individual frames as the samples for training and testing, which could have caused subsequent temporally correlated frames to end up in the train and test splits, giving overly optimistic results, we first shuffled all the frames at each of the stress levels (after combining the data from the two sessions). We then created as many non-repeating blocks as possible at each stress level by averaging 3, 4, or 5 frames[^8-11^](#_ENREF_8) and tested the three ML algorithms for each of the three block sizes, separately. We chose these three block sizes to adequately capture the slow hemodynamic response. Next, we split the data into training and testing sets with 80% and 20% of data, respectively, in a randomized, stratified fashion (i.e., the training and testing sets were randomly chosen while still preserving the percentage of [block-averaged] samples for each of the eight classes: Fig. SM1). The *train_test_split* class of the *model_selection* module of the *sklearn* library[^12^](#_ENREF_12) of Python 3.7.10 was used for this purpose[^10^](#_ENREF_10). The features from the training set were normalized by removing the mean and scaling to unit variance using the *StandardScaler* class of the pre- processing module of the *sklearn* library. The features from the testing set were then normalized using the values adopted to normalize the training set. This process of creating train-test splits was repeated 25 times for each model. Root mean square error (RMSE) was used as the performance metric for all the three ML algorithms because of the class imbalance; it punishes large errors and results in a score in the same units as the classified data. Using RMSE, as opposed to accuracy, allows for a common metric to compare the performance of classification and regression algorithms. However, given the popularity of using accuracy scores for classification tasks, we provide that metric as well, at the overall subject level for the SVM and DL approaches.

SVM models were trained using the *svc* (i.e., support vector classifier) class of the *svm* module of the *sklearn* library using Radial Basis Function (RBF) kernels[^10^](#_ENREF_10). The optimal values of the regularization parameter, C (10, 100, 1000), the kernel coefficient, γ (10^-5^, 10^-4^, 10^-3^), and the number of features (1600, 2400, 4700) were found by the *GridSearchCV* class of the *model_selection* module of the *sklearn* library. For feature selection, the *SelectKBest* class of the *feature_selection* module of the *sklearn* library was used to select features in the training data using F scores from an analysis of variance [ANOVA; ^10^](#_ENREF_10) based on linear regression of stress level by each feature. The best parameters were optimized by cross-validated grid-search over the parameter grid by further splitting the training data into train and validation sets (2:1). The model with the optimized parameters was then applied to the test set to assess performance. The approach and parameter grid space used for SVR models was the same as that for SVM models. The *svr* class of the *svm* module of the *sklearn* library was used with an RBF kernel for training all SVR models.

The approach used for training DL models was more complex. Fig. SM2 gives the general architecture of our DL model, which consisted of eight hidden perceptron layers (with a different number of nodes or “neurons” each), one input (with N nodes corresponding to N features), and one output layer (with 8 outputs, one for each class). Each of the perceptron layers consisted of a fully connected layer (i.e., each node of one layer is connected with all the nodes of the following layer), followed by a batch normalization layer[^13^](#_ENREF_13), a dropout layer, and an activation function (*reLu*: rectified linear unit) leading to the output features. The output (last layer) of each DL model used *softmax* as the activation function, with eight output nodes corresponding to the possible eight stress levels. At the end of this process, the model outputs a probability for each of the eight classes. The class with the highest probability was chosen as the predicted class. The connection between the different perceptron layers is characterized by weights, which were adjusted by a stochastic backpropagation algorithm. The *adam* optimizer[^14^](#_ENREF_14)^,^ [^15^](#_ENREF_15) was used (one of the most popular adaptive learning rate optimization algorithms designed specifically for training DL networks) with *categorical_crossentropy* as the loss function. A momentum[^16^](#_ENREF_16)^,^ [^17^](#_ENREF_17) value of 0.9 was used to aid in convergence during training. The number of hidden layers and the number of nodes in each of them were obtained by trial and error by minimizing the *categorical_crossentropy* loss. To control for overfitting, a combination of different values of dropout (0, 0.2, 0.4)[^18^](#_ENREF_18) and l2_param (0, 10-5, 10-3)[^19^](#_ENREF_19) were explored, and the best combination (which had the smallest RMSE on validation set performance) was selected for each iteration. All DL models were implemented in Python 3.7.10 using Keras 2.4.3 wrapper[^20^](#_ENREF_20) with TensorFlow 2.4.1 backend. A total of 2000 epochs were trained, with a batch size of 128. *EarlyStopping* callback was used to stop the training if there was no reduction in validation loss (validation data was created by keeping *validation_split* parameter as 0.2) for 250 epochs.

**Figure SM2.** The architecture of the multi-layer perceptron deep learning (DL) model, with the architecture of each of the perceptron layers.


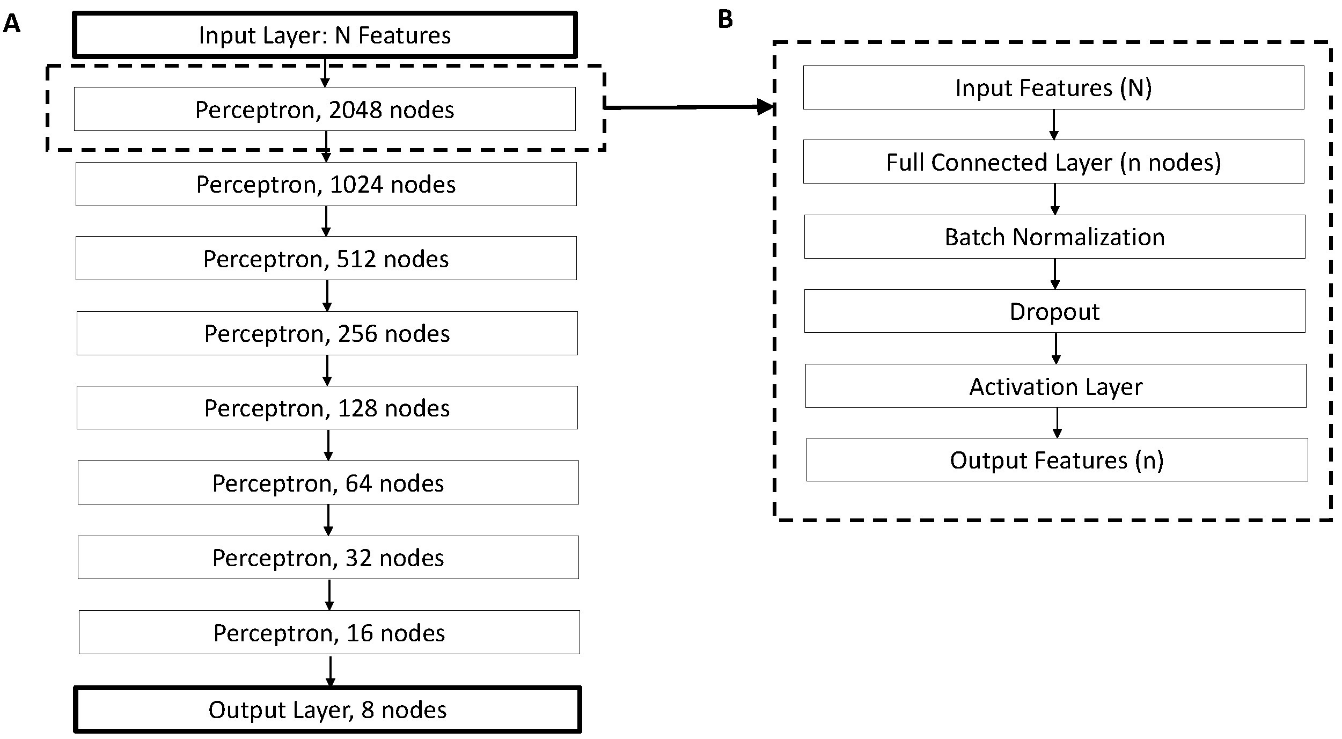


For the DL approach, since there is no *GridSearchCV* equivalent in which we could have built-in our feature selection, each of the 25 train-test split iterations was evaluated for three different numbers of features (1600, 2400, 4700) separately, using a nested approach, optimizing for other hyperparameters (*dropout and l2_param*) as mentioned above. The group of features that led to the best performance (smallest RMSE) on the validation data was selected for that iteration.

Overall, for each Veteran, after combining the data for the two sessions, we carried out 25 iterations for each of the three block sizes (3, 4, or 5), and for each of those iterations, we trained SVM, SVR, and DL models, separately. Statistical comparisons of performance metrics were obtained from the ML algorithms using paired t-tests. Computations were performed on a custom enterprise-class Linux server containing two Xeon E5-2695 v4 CPUs with 18 cores in each (2.1 GHz clock speed), 512 GB of DDR4 ECC RAM. The operating system was Ubuntu 16.04 LTS. Data were generally mounted on a 22-TB file server consisting of six 8-TB SAS drives combined into a RAID 10 array. To increase the computation speed, most computations were performed using RAM rather than disk memory for file access by way of the shared-memory volume. Importantly, for real-time effectiveness, the preprocessing steps and decoding from a trained model were completed in an average of 1.55 s for new brain volumes over the course of a single run.

*SM2.1 Computation time*

We measured training time by running the ML algorithms 25 times with a block averaging size of 4. It took an average of ~30—45 sec to train the SVC and SVR models, and about 70 minutes to train the DL model (Means ± SD of SVM, SVR, and DL models were 45.3 ± 1.2 sec, 31.9 ± 0.9 sec, and 69 ± 6.7 min, respectively). Similarly, we measured decoding times by using the trained ML models to predict test sets 25 times. Decoding was much faster, averaging <0.4 sec for all models (Means ± SD of SVM, SVR, and DL models were 0.31 ± 0.07s, 0.29 ± 0.12 s, and 0.22 ± 0.03 s, respectively).

**Figure SM3.** Box plots illustrating the results of the permutation tests for six of the Veterans. The blue boxplots represent the RMSE values for the ML models run with the original labels, while the orange boxplots represent the RMSE values for the ML models run with permuted labels


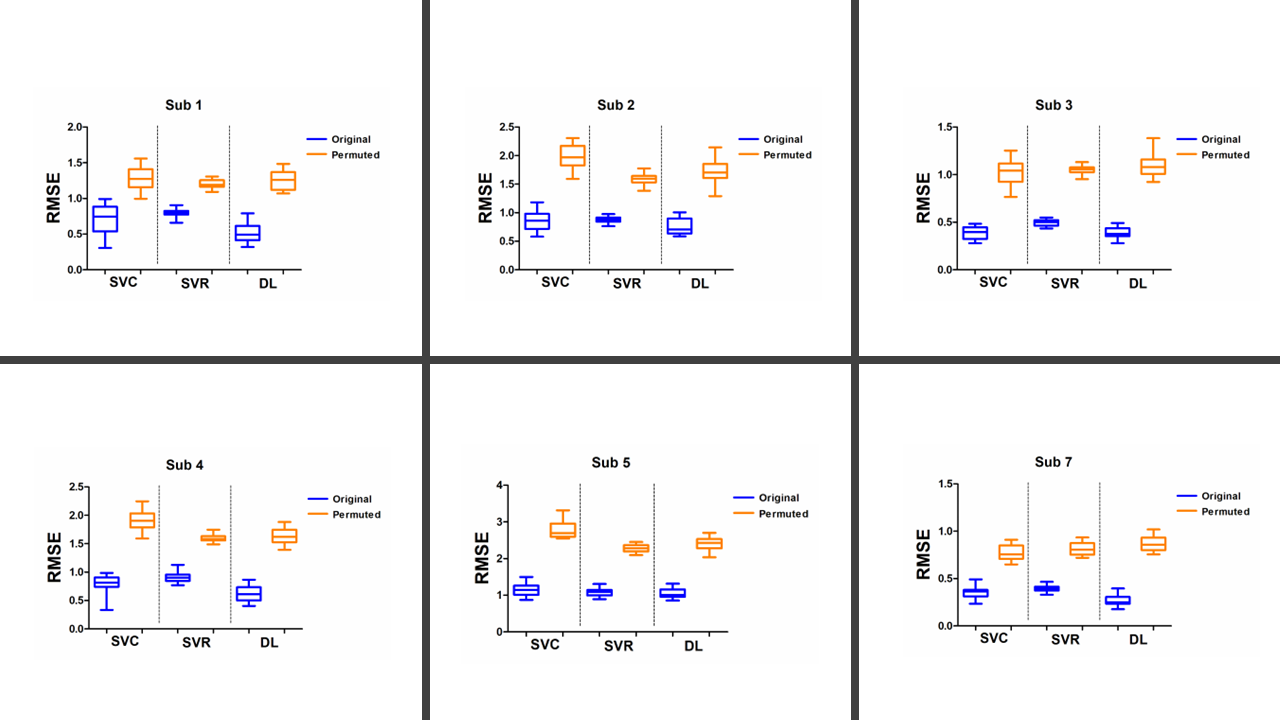


**References**

1. Roche A. A four-dimensional registration algorithm with application to joint correction of motion and slice timing in fMRI. *IEEE transactions on medical imaging*. 2011; 30: 1546-54.

2. Jenkinson M, Bannister P, Brady M and Smith S. Improved optimization for the robust and accurate linear registration and motion correction of brain images. *NeuroImage*. 2002; 17: 825-41.

3. Greve DN and Fischl B. Accurate and robust brain image alignment using boundary-based registration. *NeuroImage*. 2009; 48: 63-72.

4. Fischl B and Dale AM. Measuring the thickness of the human cerebral cortex from magnetic resonance images. *Proc Natl Acad Sci U S A*. 2000; 97: 11050-5.

5. Reuter M, Schmansky NJ, Rosas HD and Fischl B. Within-subject template estimation for unbiased longitudinal image analysis. *NeuroImage*. 2012; 61: 1402-18.

6. Naylor B, Floren A, Miikkulainen R and Ress D. Final Report: Computational Modeling of Individual Brains. In: U.S. Army Research Office, (ed.). 2019.

7. Kahn I, Desai M, Knoblich U, et al. Characterization of the functional MRI response temporal linearity via optical control of neocortical pyramidal neurons. *The Journal of neuroscience : the official journal of the Society for Neuroscience*. 2011; 31: 15086-91.

8. Misaki M, Phillips R, Zotev V, et al. Brain activity mediators of PTSD symptom reduction during real-time fMRI amygdala neurofeedback emotional training. *NeuroImage Clinical*. 2019; 24: 102047.

9. Zotev V, Phillips R, Misaki M, et al. Real-time fMRI neurofeedback training of the amygdala activity with simultaneous EEG in veterans with combat-related PTSD. *NeuroImage Clinical*. 2018; 19: 106-21.

10. Floren A, Naylor B, Miikkulainen R and Ress D. Accurately decoding visual information from fMRI data obtained in a realistic virtual environment. *Frontiers in Human Neuroscience*. 2015; 9.

11. Pereira F, Mitchell T and Botvinick M. Machine learning classifiers and fMRI: a tutorial overview. *NeuroImage*. 2009; 45: S199-209.

12. Pedregosa F, Varoquaux G, Gramfort A, et al. Scikit-learn: Machine learning in python. *Journal of Machine Learning Research*. 2011; 12: 2825-30.

13. Ioffe S and Szegedy C. Batch normalization: Accelerating deep network training by reducing internal covariate shift. *International Conference on International Conference on Machine Learning*. 2015, p. 448-56.

14. Bushaev V. Adam - latest trends in deep learning optimization. Medium, 2018.

15. Diederik P and Ba J. Adam: A method for stochastic optimization. Cornell University, 2017.

16. Bushaev V. Stochastic gradient descent with momentum. Medium, 2017.

17. Qian N. On the momentum term in gradient descent learning algorithms. *Neural networks : the official journal of the International Neural Network Society*. 1999; 12: 145-51.

18. Brownlee J. A gentle introduction to dropout for regularizing deep neural networks. Machine Learning Mastery, 2018.

19. Brownlee J. Use weight regularization to reduce overfitting of deep learning models. Machine Learning Mastery, 2018.

20. Chollet F. *Deep Learning with Python*. United States of America: Manning Publications Co., 2018.
